# Fever in Children: Pearls and Pitfalls

**DOI:** 10.3390/children4090081

**Published:** 2017-09-01

**Authors:** Egidio Barbi, Pierluigi Marzuillo, Elena Neri, Samuele Naviglio, Baruch S. Krauss

**Affiliations:** 1 Institute for Maternal and Child Health IRCCS “Burlo Garofolo”, 34137 Trieste, Italy; egidio.barbi@burlo.trieste.it (E.B.); elena.neri@burlo.trieste.it (E.N.); samuele.naviglio@burlo.trieste.it (S.N.); 2 Department of Woman and Child and General and Specialized Surgery, Università degli Studi della Campania “Luigi Vanvitelli”, 80138 Naples, Italy; 3 Department of Medicine, Surgery and Health Sciences, University of Trieste, 34137 Trieste, Italy; 4 Division of Emergency Medicine, Boston Children’s Hospital, Department of Pediatrics, Harvard Medical School, Boston 02115, MA, USA; baruch.krauss@childrens.harvard.edu

**Keywords:** fever, children, serious bacterial infection, primary care, prediction rules

## Abstract

Fever in children is a common concern for parents and one of the most frequent presenting complaints in emergency department visits, often involving non-pediatric emergency physicians. Although the incidence of serious infections has decreased after the introduction of conjugate vaccines, fever remains a major cause of laboratory investigation and hospital admissions. Furthermore, antipyretics are the most common medications administered to children. We review the epidemiology and measurement of fever, the meaning of fever and associated clinical signs in children of different ages and under special conditions, including fever in children with cognitive impairment, recurrent fevers, and fever of unknown origin. While the majority of febrile children have mild, self-resolving viral illness, a minority may be at risk of life-threatening infections. Clinical assessment differs markedly from adult patients. Hands-off evaluation is paramount for a correct evaluation of breathing, circulation and level of interaction. Laboratory markers and clinical prediction rules provide limited help in identifying children at risk for serious infections; however, clinical examination, prudent utilization of laboratory tests, and post-discharge guidance (“safety netting”) remain the cornerstone of safe management of febrile children.

## 1. Introduction

Fever, a physiologic response characterized by an elevation of body temperature above normal daily variation [1], is one of the most common causes for medical consultation in children, being responsible for 15–25% of consultations in primary care and emergency departments [2,3,4]. Although fever can be concerning to parents and caregivers, the prevalence of serious infections in children is low, estimated at <1% in primary-care settings in industrialized countries [5]. However, this figure can increase up to 25% in emergency departments [6]. We performed a narrative review on the epidemiology, assessment, and management of fever in children, with the aim of providing non-pediatric physicians with up-to-date information on the approach to febrile children.

## 2. Measurement of Fever in Children

The human body core temperature is subject to variations between and within individuals. Several physiologic factors influence body temperature, including time of the day (with a nadir in the morning and a late afternoon peak) [7]; level of activity; meals; age (infants and young children generally have higher temperatures than older children) [8]; and menstrual cycle (body temperature is about 0.4 °C higher in the luteal phase compared to the follicular phase) [9]. In infants, core temperature can be as low as 36 °C during nocturnal sleep, but can rise up to 37.8 °C during active periods of the day, especially after feeding [10]. This variability precludes the identification of a single universal upper limit of normal; therefore, fever can be generally defined as a thermoregulated elevation of body temperature above normal daily variation [1]. However, for clinical and research purposes, fever is often defined as a core temperature of 38 °C or higher [11,12].

Body temperature can be measured in the axilla, rectum, mouth, skin, and ear. There are substantial differences among measurement sites [13]. Rectal temperature is considered to be the most accurate for estimating core body temperature [1,13], and is recommended by the American Academy of Pediatrics for children less than 4 years of age [14]. However, its use is discouraged by other clinical guidelines because of safety and practical issues, as well as for the physical and psychological discomfort it may cause [1,15]. Furthermore it is contraindicated in neutropenic or immunocompromised children [16]. Other measurement sites are less accurate than rectal temperature but can be used in clinical practice. Oral temperature is considered to be one of the most accurate sites, although it is on average 0.5 °C lower than rectal temperature. However, it is not suitable for children under 5 years of age, and some children may find it uncomfortable. Axillary temperature measurement can be considered a viable alternative, since it is practical and reasonably accurate, but its sensitivity is inferior to rectal temperature [1,17,18]. In newborns, axillary temperature has been found to be as reliable as rectal temperature, although values tend to be 0.25 °C–0.5 °C lower [19,20], while in older children this difference is greater, at least 0.5 °C (0.92 °C in a systematic review [21]). In clinical practice, an axillary temperature is considered to be abnormal when it is above 37.5 °C [22].

Recommendations differ on the best site for temperature measurement in children. The National Institute for Health and Care Excellence (NICE) guidelines recommend measuring body temperature in the axilla, using an electronic thermometer for infants less than 4 weeks of age and chemical dot or electronic thermometers in older children [1], while the American Academy of Pediatrics suggests rectal thermometry for children younger than 4 years of age and oral thermometry in older children [14]. The gallium-in-glass thermometer has been suggested as an alternative for axillary thermometry as it may be more accurate than digital thermometers [23]; nevertheless, it has to be maintained in place for 5 min to assure correct measurement and glass makes it unsuitable for young children. Tympanic infrared thermometers represent a possible alternative [1,24], but their sensitivity is not optimal [25], and they are not accurate in children under 3 months of age. Chemical forehead thermometers are unreliable [1,25]. Temporal artery thermometers and forehead non-contact infrared thermometers represent emerging techniques, but further studies are needed [26,27]. Finally, even though it may be subject to interobserver differences, parental report of tactile fever should never be dismissed [1]. A prospective comparison of 322 febrile children found that mothers could accurately detect fever by tactile assessment (sensitivity 84%, specificity 76%) [28].

## 3. Increased Body Temperature as a Diagnostic Sign

Any abnormal elevation of body temperature in a child should be evaluated as a potential symptom of an underlying condition [12]. Fever is present when an increase in body temperature occurs through a modification of the hypothalamic temperature set-point due to exposure to endogenous pyrogens [29]; in contrast, hyperthermia occurs when there is an increase in body temperature because of a failure of thermoregulation, either because of increased heat absorption, heat production and/or reduced ability to dissipate it [30,31]. This difference implies that hyperthermia, in contrast to fever, may have potentially severe consequences on the body, since hyperthermia does not represent a controlled physiologic phenomenon.

Hyperthermia is less common in children, compared to fever. Most cases of hyperthermia are due to environmental hyperthermia, caused by massive heat exposure, which overcomes the body’s thermoregulation, such as in the case of “forgotten baby syndrome” involving children left in cars during hot season [32]. “Heat stroke” is defined as a core temperature ≥40 °C accompanied by central nervous system dysfunction due to environmental heat exposure [30]. Young children have less efficient heat dissipation mechanisms, compared to older children and adults [31]. Other predisposing factors include conditions characterized by excessive fluids loss or that adversely affect water-electrolyte balance (e.g., gastrointestinal illness, diabetes insipidus, diabetes mellitus, cystic fibrosis, diuretics, fever); conditions associated with suboptimal sweating (spina bifida, familial dysautonomia, hypo/anhidrotic ectodermal dysplasia, Crisponi syndrome, Fabry disease); diminished thirst/water intake (cognitive impairment, young children); hypothalamic dysfunction; anorexia nervosa; and obesity [33].

Apart from environmental heat exposure, hyperthermia may be directly caused by conditions resulting in abnormal thermoregulation or increased heat production. Central nervous system conditions involving injury to the hypothalamus (either congenital or acquired) may lead to temperature dysregulation and hyperthermia (sometimes called “neurogenic” or “central fever”). Other causes include status epilepticus, thyrotoxicosis, and genetic syndromes associated with abnormal thermoregulation. Intoxication from hyperthermia-inducing drugs may result in severe hyperthermia; involved drugs include stimulating/sympathomimetic drugs (cocaine, methamphetamine, MDMA), anticholinergic drugs (e.g., antihistamines, tricyclic antidepressants), serotoninergic drugs (serotonin syndrome), and salicylates. Neuroleptic malignant syndrome is a severe idiosyncratic reaction to antipsychotic agents, but also antiemetic agents such as metoclopramide [34], characterized by altered mental status, muscular rigidity, movement disorders, hyperthermia and autonomic dysfunction [35]. Malignant hyperthermia is a rare genetic disorder (1 in 14,000 pediatric general anesthesia) associated with several forms of congenital myopathy and triggered by succinylcholine or inhalational anesthetics agents; clinical features include rapid onset of extremely high temperature (38.5–46 °C), usually heralded by masseters spasm, muscle rigidity, metabolic acidosis, and hemodynamic collapse. Specific treatment, with discontinuation of involved anesthetics, muscular relaxation with sodium dantrolene, and correction of metabolic acidosis, has dramatically reduced the mortality, once as high as 70%, to less than 5% [36,37].

Fever is the most common reason for increased body temperature in pediatric clinical practice. The most common causes of fever in children are infections; non-infectious causes include immune-mediated, inflammatory, and neoplastic conditions. When a cause for fever cannot be identified by history and physical examination it is called “fever without source” (FWS) [38]. In industrialized countries, a minority of children with FWS will have a serious bacterial infection (SBI) (mainly urinary tract infection (UTI), less commonly pneumonia, sepsis, or meningitis), while the majority will have mild, self-resolving viral illnesses [39]. Nevertheless, signs and symptoms of a viral upper respiratory infection do not reliably exclude the possibility of an associated SBI, given the possibility of co-infections. In a study of children 2 to 36 months with FWS, at least one virus (most frequently adenovirus, human herpesvirus-6, enterovirus, and parechovirus) could be identified in 76% of children in whom no other explanation for the fever was found, but also in 40% of children with SBI [40]. Therefore, detection of viral pathogens cannot be considered a discriminating factor.

Even though the height of fever does not define severity of illness by itself, there is an association with a greater likelihood of SBI for temperatures >39 °C [1]. In a prospective cohort study on more than 12,800 children presenting with febrile illness, fever >39 °C was associated with an increased risk of SBI, especially in infants under 6 months [41]. However, this cut-off still missed 82% of SBI episodes in this age group; therefore, lower temperatures cannot be considered reassuring. In a prospective series of 103 children with a temperature >41 °C, almost 50% had an SBI [42]. Temperatures above 41 °C have also been associated with a higher risk of meningitis [43]. Notably, however, children with SBI may also have a normal temperature or be hypothermic.

## 4. The Value of Associated Clinical Findings

Gathering as much information as possible in the first, hands-off, phase of the visit is pivotal. Physical signs such as pallor, mottled appearance, ashen or blue skin color, reduced activity (poor feeding, no smile, decreased response to stimuli, lethargy, weak high-pitched cry), tachypnea and tachycardia, capillary refill time >3 s, and a reduced urine output are all concerning for SBI (“red flags”) [1,38], and should prompt a through evaluation. The meaning of some of them, however, may be put in context.

**Figure 1:**
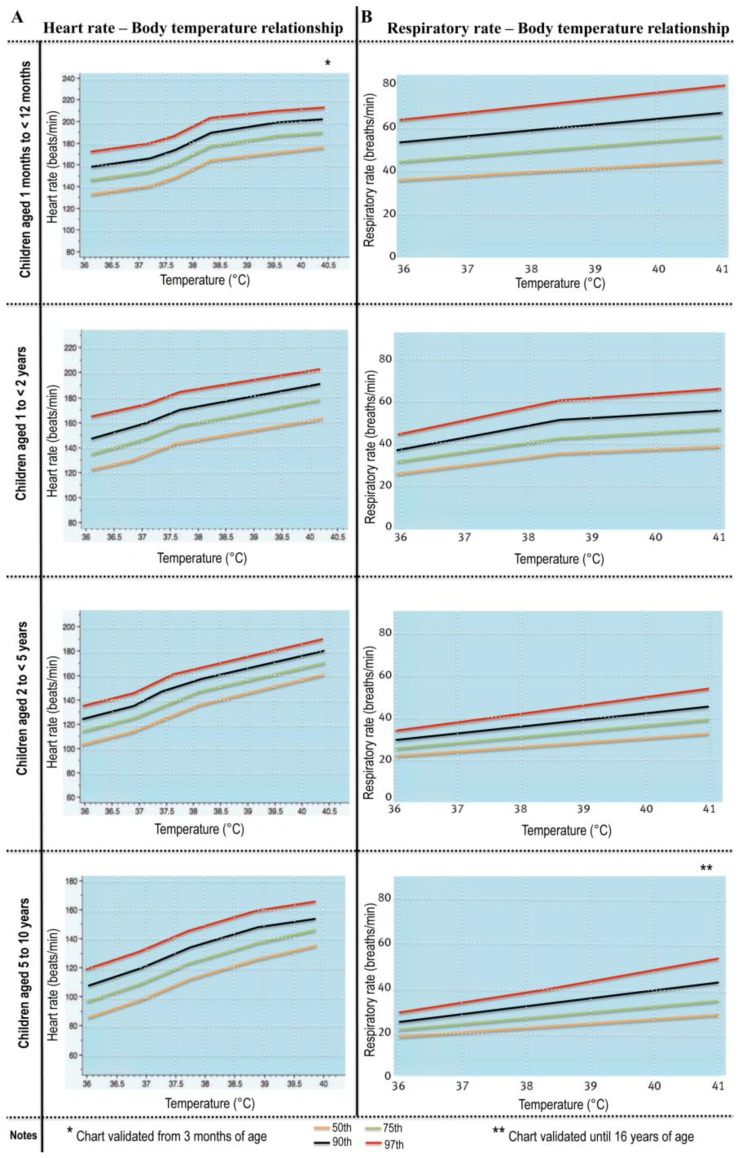
Age-specific temperature-related heart and respiratory rate centile charts (modified from references [46,47] by permission from BMJ Publishing Group Limited).

Tachypnea: although the World Health Organization criteria for the diagnosis of pneumonia include tachypnea alone [44], isolated tachypnea is a poor indicator of pneumonia in the presence of wheezing [45]. Furthermore, fever may by itself alter respiratory rate and heart rate [46,47]; therefore, using age-specific, temperature-corrected cut-offs for respiratory rate (Figure 1) has been shown to result in more accurate detection of lower tract respiratory infections than fixed thresholds [46].

Bulging fontanelle: while a bulging fontanelle can be a sign of bacterial meningitis, it may also be due to more benign causes (e.g., sixth disease). In a series of 153 febrile infants between 3 and 11 months of age with fever and a bulging fontanelle, only 1 (0.6%) had bacterial meningitis [48]. Of note, this patient also had other alarm features and leukopenia. These findings suggest that, in febrile infants with a bulging fontanelle who are otherwise well-appearing and have no laboratory evidence of bacterial infection, close observation without lumbar puncture is a reasonable option.

Non-blanching rash: although a non-blanching rash should always raise concern, well-appearing children with fever and petechiae (small, non-blanching, macular hemorrhagic skin spots <2 mm in diameter) are still at low risk of SBI [49]. In a series of 411 patients between 3 and 36 months of age, none of the 357 well-appearing children had SBI, while 6 out of 53 ill-appearing children had SBI. In another series of 55 children (mean age 2.5 years) only 9% eventually had bacterial sepsis, and they also had other concerning clinical features or abnormal laboratory tests [50]. These studies suggest that well-appearing children with fever and petechiae, without frank purpura, and with normal blood tests, can be observed for 4–6 h, reassessed, and eventually discharged.

Rigors: The presence of rigors may be associated with a higher probability of SBI (15% vs. 6% in children without rigors) [51]. Furthermore, they are also common in serious non-bacterial illness such as malaria, dengue, and chikungunya. Leg pain has also been reported as a possible early sign of bacterial sepsis and meningococcal disease [52]. Night sweats are a relatively nonspecific symptom [53]; however, their presence in the context of prolonged and unexplained febrile illness should raise concern for occult infectious (tuberculosis, endocarditis, liver and lung abscess, brucellosis) and non-infectious diseases. Finally, clinician’s intuition that “something is wrong” (i.e., “gut feeling”) has been also demonstrated to be of diagnostic value [5,54]. Gut feeling is definitely something “impalpable”; however, it likely reflects a *gestalt* evaluation of several clinical aspects that can be appreciated in the first, no touch approach to the ill child. These aspects have been further characterized and systematized in the “Pediatric Assessment Triangle” (PAT) developed by the American Academy of Pediatrics for Pediatric Advanced Life Support programs, which includes three main aspects (work of breathing, general appearance, and circulation to the skin). The PAT allows the clinician to establish the severity of the child’s condition and helps articulating the general impression of the child [55,56]. Nevertheless, gut feeling does not abolish full clinical evaluation and prudent management; therefore, its value could be to raise clinical suspicion in unclear situations rather than to forego proper standard evaluation. In conclusion, clinicians should evaluate every sign/symptom in feverish children putting it in context and should be aware that combinations of signs/symptoms could underline an increased risk of SBI. A useful classification of alarming signs/symptoms for first approach to a febrile child can be found in the NICE traffic light system [1], since it values some signs/symptoms more than others and sets a distinction between “red” and “orange” alarm features (see also the following sections).

## 5. Evaluation of Infants and Young Children with a Fever

The risk of SBI in infants <3 months of age is higher than at any other age in childhood (approximately 3.75/1000 live full term births) [57]. As a result, the prevalence of SBI in febrile infants ranges from 8% to 12.5%, and it is even higher (up to 20%) in neonates (≤28 days old) [58,59]. In this age group, clinicians cannot rely on physical examination to identify infants at risk because concerning signs and symptoms may not be apparent; therefore, in-hospital observation and laboratory testing are recommended, even in patients with signs and symptoms of a viral respiratory infection [1,38]. All children in this age group should have urinalysis to rule out UTI, complete blood count, and blood cultures, plus stool culture in cases of diarrhea and chest X-ray in cases of signs of pulmonary disease. Apart from the neonatal period, however, chest X-ray may be omitted if there is a high likelihood of uncomplicated bronchiolitis, since bronchiolitis can be by itself associated with chest radiograph abnormalities that may prompt an undue utilization of antibiotics, reserving therefore radiographs to severe cases or those in which a complication is suspected [60,61]. It should be remembered, however, that presence of Respiratory Syncytial Virus or other viruses affecting the airways does not exclude the possibility of a bacterial infection; therefore, a high clinical suspicion of SBI should be maintained [62]. Inflammatory markers like C-reactive protein (CRP) and procalcitonin (PCT) may aid in identifying children at risk for SBI. Nevertheless, even though they perform better than white blood cell count (WBC), their sensitivity and predictive ability are limited [63,64,65]. While the performance of CRP and PCT is generally similar, there is some evidence that PCT may be more accurate than CRP for detecting invasive bacterial infections (IBI, defined as bacteremia and meningitis) in children <3 months of age. A recent multicentric French prospective cohort study evaluated the diagnostic performance of PCT and CRP in a population of 2047 infants between 7 and 91 days of age admitted for fever to emergency departments. PCT and CRP had a similar diagnostic accuracy for detecting SBI, but PCT performed significantly better than CRP in infants with IBI (using a cutoff value of 0.3 ng/mL for PCT and 20 mg/L for CRP) [65]. This advantage may be associated with the fact that PCT has been shown to have a greater predictive value than CRP for IBI in the very first hours (<8 h) from fever onset [66]. However, in all other instances, CRP may still be a preferable marker to use in clinical practice, since it is similarly accurate and less expensive than PCT [1].

Clinical management is less controversial for children <1 month of age with fever, in whom lumbar puncture and hospitalization for empiric antibiotic therapy pending cultures are always recommended. In full-term children 1 to 3 months of age, there are wider variations in practice patterns. Lumbar puncture and parenteral antibiotics are always recommended for ill-appearing children [67], while in well-appearing children, observation and blood test results may be useful to identify children at higher risk [1].

For children 3 to 36 months of age there is more variability in clinical practice, and no single guideline has been universally adopted [38]. The introduction of conjugate vaccines for *Haemophilus influenzae, Streptococcus pneumoniae*, and *Neisseria meningitidis* has led to a significant reduction in SBI [68], with a reduction of bacteremia rates from 2.0–3.4% in the pre-conjugate vaccine era to 0.34% in the post-conjugate vaccine era [69,70]. This shifting epidemiology has led to a modification in the emergency department evaluation of fever, with a decline in laboratory testing [71]. However, immunization status should always be specifically questioned, and children with incomplete immunizations should be considered at greater risk. Moreover, despite a global decrease in invasive pneumococcal disease [72,73], an increased incidence of empyema and mastoiditis has been reported, possibly due to serotypic shifting towards non-vaccine strains [74,75]. This may change after the introduction of the 13-valent vaccine [73,76]; however, there is already evidence of increasing invasive pneumococcal diseases due to serotypes not included in the 13-valent vaccine [72].

UTI can be clinically inapparent in well-appearing children with FWS. UTI is the most common SBI in febrile children <24 months, with an overall prevalence of 7% (and even higher in the presence of risk factors) [77]. There is some discordance on who should be assessed for UTI: the American Academy of Pediatrics 1999 guidelines recommended that all children aged 2 to 24 months with FWS be tested for UTI. The 2011 update introduced a probability-based algorithm depending on the presence of risk factors for UTI (gender, age, ethnicity, circumcision status, height and duration of fever, and absence of other sources of infection) [78]. In clinical practices, however, recommendations are often unattended, and urine testing may not be performed, even in patients who should have it as per their pre-test characteristics [79]. The American College of Emergency Physicians maintains a simpler approach, recognizing that no clinical feature has been shown to effectively exclude UTI, and suggesting that physicians should consider urine testing in children aged 2 to 24 months, especially among those at higher risk [67]. The NICE guidelines similarly suggest that children with unexplained fever should have urine tested to exclude UTI, without specifying a pre-selection of patients.

Contrary to popular beliefs, teething is not a cause of fever [80]. Immunization, however, is a common cause, and up to 50% of infants may experience fever 24–48 h after immunizations. Prophylactic treatment with acetaminophen reduces the incidence of fever, but may transiently decrease antibody response [81]. Since there are few data on the long-term effects of this practice [82], and since many children may not need it at all, we do not suggest routine prophylactic treatment with acetaminophen after immunizations, reserving it for symptomatic children.

## 6. The Value of Clinical Prediction Rules

Many clinical prediction rules and national guidelines have been developed for the evaluation of febrile children. The most commonly used clinical prediction rules include Rochester criteria (for infants aged 0 to 60 days) [83], Philadelphia criteria (for infants aged 29–60 days) [84], and Boston criteria (for infants aged 28–89 days) [85]. These were designed to provide a set of reassuring criteria that allow safe discharging if requirements are met. Overall, these criteria tend to have a very high negative predictive value (over 99%) but a low positive predictive value (14% for Philadelphia criteria, 12.3% for Rochester criteria). In fact, they were designed to have a high sensitivity for SBI (i.e., allow safe discharge), even though at expenses of specificity, and they still represent the basis of standard clinical management of febrile infants in many institutions.

Further models were developed to try to improve diagnostic performance, resulting in many diverse clinical prediction rules. Their performance in clinical practice, however, has often been shown to be suboptimal [86]. This may be due in part to the complexity and diversity of bacterial infections, which may be difficult to recapitulate entirely in predetermined models. Furthermore, many clinical prediction rules have limited or no external validation [86]. A meta-analysis comparing four clinical prediction rules (Five-Stage Decision Tree, Yale Observation Scale, a pneumonia rule, and a meningitis rule) and two national guidelines (the 2007 NICE traffic light system and the Dutch College of General Practitioner Guidelines) showed that none provided perfect diagnostic accuracy [87]. However, the NICE and the Dutch College of General Practitioner guidelines were very effective in ruling out SBI, but with a substantial proportion of false-positive cases. In fact, a low predictive value for ruling-in SBI has been demonstrated for many of the “red features” of the 2013 NICE guidelines traffic light system [88]. These guidelines were developed to maximize sensitivity rather than specificity, in order not to miss SBI even in low prevalence settings; however, further validation may be necessary before widespread adoption. A recent multicenter study evaluated the performance of Yale Observation Scale in a prospective cohort of 4591 non–critically ill, febrile, full-term infants ≤60 days of age. Of the 4058 infants with Yale Observation Scale scores ≤10, 388 (9.6%) had SBIs (sensitivity: 11.6%; negative predictive value: 90.4%) and 72 (1.8%) had invasive bacterial infections (sensitivity 24.2%; negative predictive value 98.2). Notably, even though performance was suboptimal for both, the Yale Observation Scale was actually outperformed by the unstructured risk evaluation given by the attending physician: among infants for whom clinician suspicion for SBI was estimated to be <1%, only 106 had SBIs (6.4%) and 16 (1.0%) had invasive bacterial infections. One of the best performing models so far (actually outperforming physicians’ assessment) was developed in Australia using on a computer system integrating a larger number of clinical variables [89]. This approach, however, may be difficult to implement in clinical practice. Prediction rules integrating laboratory tests may represent a useful adjunct; however, their implementation also requires deciding which children need a venipuncture. Recently, however, a clinical model integrating CRP and clinical features has been shown to perform no better than usual care in a pediatric emergency department [90].

Considering the limits of the existing clinical guidelines and prediction rules, as well as the possibility of unpredictable progression of illness, careful clinical examination, watchful waiting, prudent utilization of laboratory tests, and post-discharge guidance (safety netting strategies, especially parental education) remain the cornerstone of safe management of febrile children [91]. Recent evidence suggests that well-looking children have a low probability of poor outcome even in case of unpredictable progression toward a severe illness. In a series of 521 children with meningitis and sepsis, there was no difference in outcome (mortality, critical care use, and length of hospital stay) in children who had been “missed” at the first evaluation [92]. The most likely explanation for this finding is that most of them appeared “well-looking” at the time of the first evaluation because they were in fact affected by some minor infectious disease, and they developed sepsis or meningitis only thereafter. This suggests that, beyond the neonatal period, sepsis and meningitis are evident conditions (“*sick kids look sick*”) [93], and that clinicians should continue to rely on their diagnostic skills, provided they educate parents about signs and symptoms that should prompt urgent re-evaluation.

The risk of severe illness in febrile children is higher in developing countries [94], as well as in children with co-morbidities (e.g., cancer, immune deficiencies, neurodevelopmental disabilities, sickle cell disease, children with central venous catheters, etc.) [95,96]; therefore the approach should be modified.

## 7. Fever in Children with Disability and Cognitive Impairment

Fever in children with cerebral palsy, cognitive impairment or disability can represent a clinical challenge, since these children may have specific vulnerabilities, reduced communication abilities and are more difficult to evaluate.

Pneumonia is the most frequent cause of severe infections and death in this population [97]. Pulmonary infections are mainly caused by aspiration, which can be clinically silent, caused by dysphagia, pooling of secretions in the oropharynx, gastro-esophageal reflux, and inappropriate feeding techniques. Infections may be complicated by limited cough efficacy, restrictive lung disease, colonization by multidrug resistant pathogens, secondary bronchiectasis, and poor nutritional status [97]. These children are also at risk of severe viral respiratory infections and should receive yearly influenza immunization [98]. Proper acute management includes collecting sputum samples and antibiotic treatment to include common community-acquired microorganisms and anaerobes. First line treatment failure or previous isolation of opportunistic organisms, such as *Pseudomonas* spp., should prompt specific antibiotic treatment. Prophylactic measures include full immunization, including anti-pneumococcal vaccination, attempts to eradicate *Pseudomonas* at first isolation, and nebulized antibiotic therapy (colistin or tobramycin) in the case of serial isolation. Prophylactic oral antibiotic treatment may be considered in case of more than three episodes of pneumonia per year.

Children with cerebral palsy are also more prone to urinary tract infection, being the second cause of infection in a series with a prevalence of 13% [99]. Possible reasons include lower urinary tract dysfunction, incomplete bladder emptying, detrusor hyperreflexia and detrusor sphincter dyssynergia, vesicoureteral reflux, and the inability to communicate bladder fullness and the need to void [100]. These factors, together with an impaired mobility and a high prevalence of constipation, may all explain the increased risk for urinary retention. Furthermore, these infections are often caused by multi-resistant microorganisms [101]. Finally, these patients are at a high risk for caries and infection due to poor oral hygiene, lack of proper tongue movements, drooling, special diets, and lack of routine dental care [99,102]. Dental infections may be a cause of fever and/or pain without source in this population until this possibility is taken into consideration.

When evaluating febrile children with cognitive impairment, the child’s specific disabilities should be considered, and a complete review of organ systems should be undertaken. In addition to from the previously mentioned causes of infection, these children are also at risk for infectious complications to due to pressure ulcers, orthopedic prostheses, ventriculoperitoneal shunts, or other device malfunctioning [103]. Beside infections, other causes to consider include impaired central temperature-regulating mechanisms (central fever), hyperthermia from severe dystonia with elevated creatine phosphokinase levels [104], medications (e.g., anticholinergic drugs) [105], or their withdrawal (e.g., baclofen withdrawal syndrome) [106]. Malnutrition is also frequently observed, and may facilitate recurrent infections; cases of fever due to scurvy have also been reported [107]. Finally, these children have an increased risk of dehydration and heat-related illness [103]. Dehydration, in turn, may increase the risk of adverse effects from antipyretic drugs, while the concomitant use of anticonvulsants may increase the risk of acetaminophen-induced hepatic toxicity [108,109].

## 8. Recurrent Fevers

Respiratory infections represent the main cause of recurrent fevers in young children, and there is a great variability in the number of infections per year. A normal infant may experience up to 11 respiratory infection episodes per year, especially if they have older siblings or attend daycare centers [110]. Most of these episodes are due to self-limiting viral infections, and these children do not have other alarm features suggesting an immune deficiency disorder. On the other hand, unusually frequent serious infections (e.g., 2 or more pneumonias or deep-seated infections or sinus infections within a year; 8 or more episodes of otitis media within a year), infections requiring unusually long treatment for recovery, as well as infections by unusual or opportunistic pathogens, should raise concern for an immune deficiency disorder [111].

Several non-infectious conditions can cause recurrent fevers, which may present in a periodic pattern. PFAPA (periodic fever, aphthous stomatitis, pharyngitis, and cervical adenitis) syndrome is the most common cause of periodic fever in children, and is diagnosed on clinical criteria (regularly recurrent bouts of fever lasting 3–6 days with onset before 5 years of age, and at least 1 of the 3 associated symptoms, in the absence of upper respiratory tract infections or cyclic neutropenia) [112]. Fever episodes show a dramatic response to a single dose of oral corticosteroids. Monogenic autoinflammatory diseases, such as familial Mediterranean fever, mevalonate kinase deficiency, tumor necrosis factor receptor-associated periodic syndrome, and cryopyrin-associated periodic syndromes, are also characterized by periodic fevers, often associated with other signs and symptoms, including cutaneous rash, involvement of serous membranes (peritonitis, pericarditis), eye (periorbital pain or edema, conjunctivitis, uveitis, keratitis), muscle, joint, and nervous system (cranial neuropathies, hearing loss). Since their clinical presentation may overlap with PFAPA, a clinical score based on family history, age at onset (the younger the onset, the more likely it is genetic), presence of diarrhea, abdominal or thoracic pain, and absence of aphthosis, has been developed to identify patients with greater probability of carrying genetic mutations for these disorders [112]. It should be noted, however, that early in life, familial Mediterranean fever may begin with an atypical presentation characterized by attacks of fever alone, possibly delaying diagnosis [113]. Recurrent unexplained fever in infants in the absence of diaphoresis should raise concern for diabetes insipidus, Crisponi syndrome, familial dysautonomia, or hypohidrotic ectodermal dysplasia [114]. Recurrent episodes of fever associated with arm and leg pain may indicate Fabry disease [115].

## 9. Fever of Unknown Origin

Fever of unknown origin (FUO) in children is defined as a fever lasting more than 1 week with negative preliminary investigations. Infections are the most frequent cause of FUO (51% in a series) [116], followed by non-infectious causes (25%), with 23% remaining unexplained. Some causes of FUO are of particular importance in childhood and should be remembered in perplexing cases.

Hemophagocytic lymphohistiocytosis (HLH) is a life-threatening inflammatory syndrome resulting from an excessive and uncontrolled activation of the immune system that may be due to a genetic disorder or secondary to acquired conditions, both infectious and non-infectious [117]. It most frequently affects infants <2 years of age but may occur at all ages. Delays in the diagnosis are among the main causes of poor outcomes. HLH should be suspected in cases of unexplained fever with multiple organ involvement (most commonly hepatomegaly, splenomegaly, lymphadenopathy, but also central nervous system, respiratory, cardiac, and skin involvement) associated with specific laboratory abnormalities (cytopenias, high serum ferritin levels, liver function and coagulation abnormalities, low serum fibrinogen, hypertriglyceridemia) [118]. A useful red flag is represented by the fall of the erythrocyte sedimentation rate despite worsening of patient’s conditions. Visceral leishmaniasis should also be considered in this context [119].

Factitious fever should be suspected in school-age children and adolescents with a history of persistent fever without skin warmth or sweating, normal clinical and laboratory findings, prolonged loss of school days, and a normal temperature when not self-measured [120]. Similarly, caregiver-fabricated illness (also known as Munchausen-by-proxy syndrome), a form of child abuse, should be considered in cases of unexplained prolonged fevers or infectious symptoms [121]. Illness may be falsely reported, simulated, or actually induced. Common presenting features include recurrent fevers, infections, diarrhea, and rashes, often with a history of multiple diagnostic procedures, medical treatments, and hospitalizations. Typically, despite extensive investigations, the presented complaints cannot convincingly be attributed to known medical conditions, and the child often improves in the absence of the caregiver, who, notably, may not seem to be concerned about the patient’s symptoms.

Drug fever is a febrile reaction to a specific drug in the absence of other conditions that could explain it (Table 1).

Typically, fever disappears once the offending drug is discontinued [122]. Drug fever can be difficult to diagnose during an intercurrent infectious disease, since fever could be misinterpreted as a sign of the underlying disease.

**Figure 2:**
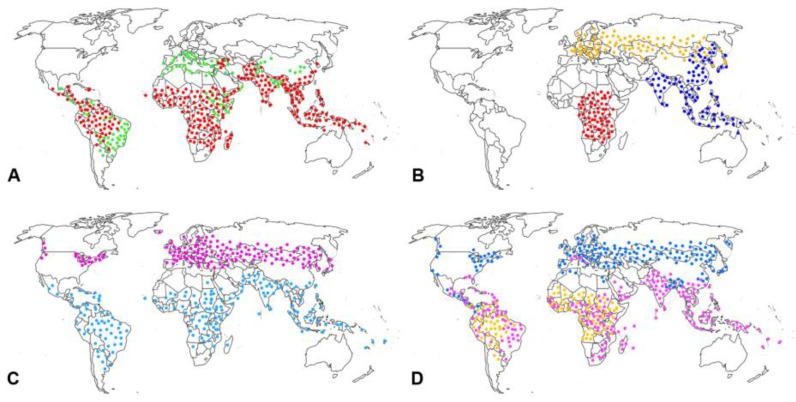
Geographical distribution of some of the most frequent infectious diseases to be considered in feverish children returning from international travels. Dots outline the area of geographical distribution of the disease; they do not indicate single locations or relative incidence (**A**) Red: malaria. Green: visceral leishmaniasis. (**B**) Red: African trypanosomiasis. Yellow: tick-borne encephalitits. Blue: Japanese encephalitis. (**C**) Blue: dengue. Violet: Lyme’s disease. (**D**) Blue: babesiosis. Yellow: yellow fever. Violet: chikungunya (data from [123]).

In investigating FUO, a complete history can be extremely useful, and should include contact with infected individuals or animals, previous travel history (Figure 2) [123], alimentary habits, drugs, bites from animals, ticks, or insects, as well as past medical history. All unnecessary drugs should be discontinued.

## 10. Treatment of Fever

Fever is one of the most worrisome symptoms for parents and caregivers [124], who are frequently concerned that untreated fever may lead to brain damage, seizures and death, despite evidence to the contrary [125,126]. Similar concerns have been reported among healthcare providers [127]. The term “fever phobia” has been used to refer to anxiety and misconceptions about fever [128]. While the central nervous system is sensitive to extreme temperatures (over 41.5 °C) [129], fever represents a controlled physiologic phenomenon, and temperatures over 41 °C are remarkably rare, possibly owing to protective mechanisms in the thermoregulatory centers [42,43]. Adverse events following a febrile illness are therefore related to the underlying condition rather than to the rise in temperature [126]. Paradoxically, the most serious and common adverse events associated with fever are related to antipyretic drugs [130].

Fever plays a physiologic role in response to infection, inhibiting bacterial growth and viral replication, and enhancing the immune response [131]. A recent meta-analysis, however, found no evidence that use of antipyretics prolongs illness in children [132]. Nevertheless, since fever itself is not dangerous, antipyretic treatment should be reserved for distressed children, aiming at improving the child’s wellbeing rather than achieving normothermia. Antipyretic treatment has not been shown to prevent recurrence of febrile seizures [133] and should therefore not be recommended for this purpose.

Response to antipyretics cannot predict the severity of the underlying illness, since children with bacterial and viral illnesses have a similar response to antipyretics [134]. However, evaluating if the child’s conditions markedly improve with antipyretic treatment may be useful to discern whether it was related to fever or to the severity of the underlying illness [135]. Parents should be instructed to observe for signs and symptoms of serious illness or dehydration in the child, rather than concentrate solely on temperature.

Fever management may differ in specific clinical situations. In children with inherited metabolic and mitochondrial diseases, catabolic stressors should be avoided, and both fever and underlying infections should be treated [136]. Fever may increase metabolic and oxygen consumption; therefore, aggressive treatment may be more important in children with a limited cardiopulmonary or metabolic reserve [135], and it is recommended in patients recovering from cardiac arrest [137].

Physical treatments like tepid sponging or cold baths are not recommended, since their efficacy is modest and they can distress the child. Similarly, undressing or over-dressing are not recommended in order to avoid excessive shivering or over-heating [1]. Ibuprofen and acetaminophen are the only drugs approved for treatment of fever in children [1,135], and they are generally considered to be equally safe and effective for reducing temperature and relieving discomfort. Ibuprofen, at 10 mg/kg/dose, may provide a more rapid and longer lasting effect than acetaminophen, but this difference was less evident in studies evaluating acetaminophen at 15 mg/kg rather than at 10 mg/kg [138]. The use of higher doses of acetaminophen as a loading dose or when administered rectally is not recommended [135].

Combination therapy with acetaminophen plus ibuprofen seems to be slightly more effective in reducing body temperature compared with monotherapy alone [139]. Nonetheless, it does not seem to provide better results for discomfort, which should be the primary aim of treatment, and it may increase medication errors and adverse events and is therefore discouraged [1]. Furthermore, an increased risk of kidney injury in children on combination therapy has been reported [140]. Alternating acetaminophen and ibuprofen may be considered only if the drug previously given has not reduced the child’s distress or if distress recurs before the next dose is due [1]. Parents’ use of antipyretics is often incorrect, both in term of dosing (including the adoption of inaccurate unit of measurement, such as “a spoon”) and frequency [141]; therefore, instructions should always be reviewed.

## 11. Adverse Effects of Fever Treatment

Both ibuprofen and acetaminophen are considered safe when used appropriately, and adverse events are rare. The most serious adverse effects are hepatic injury for acetaminophen, and acute kidney injury and gastrointestinal bleeding for ibuprofen. Errors in medications dose or frequency of administration are often implicated. Use of adult preparations has been shown to be especially dangerous [142]. Adverse events may also occur at correct dosing, especially in the presence of risk factors (Table 2), most commonly dehydration, or in the case of protracted therapies [143,144].

An increased asthma risk in early childhood from acetaminophen and ibuprofen use has been reported, but this association remains questionable after adjusting for respiratory infections [145].

## 12. Conclusions

Evaluation and management of fever in children may be improved by appropriate clinical practices. Future studies will need to focus on the evaluation and comparison of the most effective techniques for temperature measurement in children as well as on implementing evidence-based practice for evaluation of feverish children. The value and cost effectiveness of existing clinical prediction rules and guidelines in determining the risk of serious illness in febrile children should be better assessed, especially regarding the characterization of what makes clinicians suspect that “something is wrong”. Finally, studies integrating both in-hospital and post-discharge phases of children assessment are needed, especially evaluating the reliability of parents in assessing the progression of illness and the efficacy of safety-netting strategies.

## Figures and Tables

**Table 1:** Mechanisms of drug fever in children. (Modified from [122]).

Mechanism	Drugs
Altered thermoregulation	Antihistamines, antileukotrienes, atropine, levothyroxine, monoamine oxidase inhibitors, phenothiazines, epinephrine
Administration related	Amphotericin B, bleomycin, cephalosporins, vaccines, vancomycin
Pharmacologic action of the drug	Anti-neoplastic agents (e.g., 6-mercaptopurine, bleomycin, chlorambucil, cisplatin, cytosine arabinoside, L-asparaginase, vincristine), heparin, sirolimus, everolimus.
Idiosyncratic reaction	Anesthetic agents (e.g., enflurane, halothane) chloramphenicol, haloperidol, phenothiazines, nitrofurantoin, primaquine phosphate, quinidine, quinine, sulphonamides
Hypersensitivity reaction	Allopurinol, antimicrobial agents, carbamazepine, phenytoin, procainamide, quinidine, quinine, sulphonamides

**Table 2:** Risk factors and contraindications for antipyretic drugs in children (from [116,117,118,119,120,121,122]).

Risk Factors			
Ibuprofen			Acetaminophen
Gastrointestinal Complications	Renal Injury		Hepatotoxicity
Previous peptic ulcer High dose or multiple NSAIDs use Concomitant corticosteroid therapy Concomitant anticoagulant therapy	High dose Volume depletion Low urine output Concomitant use of diuretics, ACE inhibitors, sartans Concomitant administration of acetaminophen		Diabetes mellitus Obesity Chronic undernutrition or prolonged fasting Myopathies Protracted therapy. Concomitant therapy with antiepileptic drugs, isoniazid, rifampin, trimethoprim-sulfamethoxazole.
**Contraindications**			
**Ibuprofen**		**Acetaminophen**	
Allergy, angioedema or bronchospasm reactivity to NSAIDs Volume depletion Renal impairment Hepatic impairment/portal hypertension Duct-dependent congenital heart diseases Thrombocytopenia or clotting disorders Active peptic ulcer disease Inflammatory bowel diseases		Allergy, angioedema or bronchospastic reactivity to acetaminophen Severe hepatic impairment	

NSAIDs: Non-steroidal anti-inflammatory drugs; ACE: Angiotensin Converting Enzyme.

## References

[1] National Collaborating Centre for Women’s and Children’s Health (2013). Feverish Illness in Children: Assessment and Initial Management in Children Younger than 5 Years.

[2] Whitburn S., Costelloe C., Montgomery A.A., Redmond N.M., Fletcher M., Peters T.J., Hay A.D. (2011). The frequency distribution of presenting symptoms in children aged six months to six years to primary care. Prim. Health Care Res. Dev..

[3] De Bont E.G.P.M., Lepot J.M.M., Hendrix D.A.S., Loonen N., Guldemond-Hecker Y., Dinant G.-J., Cals J.W.L. (2015). Workload and management of childhood fever at general practice out-of-hours care: An observational cohort study. BMJ Open.

[4] Sands R., Shanmugavadivel D., Stephenson T., Wood D. (2012). Medical problems presenting to paediatric emergency departments: 10 years on. Emerg. Med. J..

[5] Van den Bruel A., Aertgeerts B., Bruyninckx R., Aerts M., Buntinx F. (2007). Signs and symptoms for diagnosis of serious infections in children: A prospective study in primary care. Br. J. Gen. Pract..

[6] Nijman R.G., Vergouwe Y., Thompson M., van Veen M., van Meurs A.H.J., van der Lei J., Steyerberg E.W., Moll H.A., Oostenbrink R. (2013). Clinical prediction model to aid emergency doctors managing febrile children at risk of serious bacterial infections: Diagnostic study. BMJ.

[7] Mackowiak P.A., Wasserman S.S., Levine M.M. (1992). A critical appraisal of 98.6 degrees F, the upper limit of the normal body temperature, and other legacies of Carl Reinhold August Wunderlich. JAMA.

[8] Iliff A., Lee V.A. (1952). Pulse Rate, Respiratory Rate, and Body Temperature of Children between Two Months and Eighteen Years of Age. Child Dev..

[9] Baker F.C., Driver H.S. (2007). Circadian rhythms, sleep, and the menstrual cycle. Sleep Med..

[10] Anderson E.S., Petersen S.A., Wailoo M.P. (1990). Factors influencing the body temperature of 3–4 month old infants at home during the day. Arch. Dis. Child..

[11] National Institute for Health and Care Excellence Clinical Knowledge Summaries Feverish Children—Management. http://cks.nice.org.uk/feverish-children-management.

[12] Nield L.S., Kamat D.F., Kliegman R.M., Stanton B.F., St Geme J.W.I., Schor N.F. (2015). Nelson Textbook of Pediatrics.

[13] Taylor N.A.S., Tipton M.J., Kenny G.P. (2014). Considerations for the measurement of core, skin and mean body temperatures. J. Therm. Biol..

[14] American Academy of Pediatrics (2012). Fever and Your Child.

[15] El-Radhi A.S. (2014). Determining fever in children: The search for an ideal thermometer. Br. J. Nurs..

[16] Freifeld A.G., Bow E.J., Sepkowitz K.A., Boeckh M.J., Ito J.I., Mullen C.A., Raad I.I., Rolston K.V., Young J.-A.H., Wingard J.R. (2011). Infectious Diseases Society of America Clinical practice guideline for the use of antimicrobial agents in neutropenic patients with cancer: 2010 update by the infectious diseases society of america. Clin. Infect. Dis..

[17] Falzon A., Grech V., Caruana B., Magro A., Attard-Montalto S. (2003). How reliable is axillary temperature measurement?. Acta Paediatr..

[18] Niven D.J., Gaudet J.E., Laupland K.B., Mrklas K.J., Roberts D.J., Stelfox H.T. (2015). Accuracy of peripheral thermometers for estimating temperature: A systematic review and meta-analysis. Ann. Intern. Med..

[19] Charafeddine L., Tamim H., Hassouna H., Akel R., Nabulsi M. (2014). Axillary and rectal thermometry in the newborn: Do they agree?. BMC Res. Notes.

[20] Smith J., Alcock G., Usher K. (2013). Temperature measurement in the preterm and term neonate: A review of the literature. Neonatal Netw..

[21] Craig J.V., Lancaster G.A., Williamson P.R., Smyth R.L. (2000). Temperature measured at the axilla compared with rectum in children and young people: Systematic review. BMJ.

[22] Van den Bruel A., Aertgeerts B., De Boeck C., Buntinx F. (2005). Measuring the body temperature: How accurate is the Tempa Dot?. Technol. Health Care.

[23] Schreiber S., Minute M., Tornese G., Giorgi R., Duranti M., Ronfani L., Barbi E. (2013). Galinstan thermometer is more accurate than digital for the measurement of body temperature in children. Pediatr. Emerg. Care.

[24] Batra P., Goyal S. (2013). Comparison of rectal, axillary, tympanic, and temporal artery thermometry in the pediatric emergency room. Pediatr. Emerg. Care.

[25] Teller J., Ragazzi M., Simonetti G., Lava S. (2014). Accuracy of tympanic and forehead thermometers in private paediatric practice. Acta Paediatr..

[26] Teran C.G., Torrez-Llanos J., Teran-Miranda T.E., Balderrama C., Shah N.S., Villarroel P. (2012). Clinical accuracy of a non-contact infrared skin thermometer in paediatric practice. Child. Care. Health Dev..

[27] Reynolds M., Bonham L., Gueck M., Hammond K., Lowery J., Redel C., Rodriguez C., Smith S., Stanton A., Sukosd S. (2014). Are temporal artery temperatures accurate enough to replace rectal temperature measurement in pediatric ED patients?. J. Emerg. Nurs..

[28] Graneto J.W., Soglin D.F. (1996). Maternal screening of childhood fever by palpation. Pediatr. Emerg. Care.

[29] Sessler D.I., Miller R.D., Eriksson L.I., Fleisher L.A., Wiener-Kronish J.P., Cohen N.H., Young W.L. (2015). Temperature Regulation and Monitoring. Miller’s Anesthesia.

[30] Bouchama A., Knochel J.P. (2002). Heat stroke. N. Engl. J. Med..

[31] Bytomski J.R., Squire D.L. (2003). Heat illness in children. Curr. Sports Med. Rep..

[32] Ferrara P., Vena F., Caporale O., Del Volgo V., Liberatore P., Ianniello F., Chiaretti A., Riccardi R. (2013). Children left unattended in parked vehicles: A focus on recent italian cases and a review of literature. Ital. J. Pediatr..

[33] Bergeron M.F., Devore C., Rice S.G., Council on Sports Medicine and Fitness and Council on School Health (2011). American Academy of Pediatrics Policy statement—Climatic heat stress and exercising children and adolescents. Pediatrics.

[34] Lau Moon Lin M., Robinson P.D., Flank J., Sung L., Dupuis L.L. (2016). The Safety of Metoclopramide in Children: A Systematic Review and Meta-Analysis. Drug Saf..

[35] Ghaziuddin N., Hendriks M., Patel P., Wachtel L.E., Dhossche D.M. (2017). Neuroleptic Malignant Syndrome/Malignant Catatonia in Child Psychiatry: Literature Review and a Case Series. J. Child Adolesc. Psychopharmacol..

[36] Rosenberg H., Davis M., James D., Pollock N., Stowell K. (2007). Malignant hyperthermia. Orphanet J. Rare Dis..

[37] Bamaga A.K., Riazi S., Amburgey K., Ong S., Halliday W., Diamandis P., Guerguerian A.-M., Dowling J.J., Yoon G. (2016). Neuromuscular conditions associated with malignant hyperthermia in paediatric patients: A 25-year retrospective study. Neuromuscul. Disord..

[38] Arora R., Mahajan P. (2013). Evaluation of child with fever without source: Review of literature and update. Pediatr. Clin. N. Am..

[39] Manzano S., Bailey B., Gervaix A., Cousineau J., Delvin E., Girodias J. (2011). Markers for bacterial infection in children with fever without source. Arch. Dis. Child..

[40] Colvin J.M., Muenzer J.T., Jaffe D.M., Smason A., Deych E., Shannon W.D., Arens M.Q., Buller R.S., Lee W.-M., Weinstock E.J.S. (2012). Detection of viruses in young children with fever without an apparent source. Pediatrics.

[41] De S., Williams G.J., Teixeira-Pinto A., Macaskill P., McCaskill M., Isaacs D., Craig J.C. (2015). Lack of Accuracy of Body Temperature for Detecting Serious Bacterial Infection in Febrile Episodes. Pediatr. Infect. Dis. J..

[42] Trautner B.W., Caviness A.C., Gerlacher G.R., Demmler G., Macias C.G. (2006). Prospective evaluation of the risk of serious bacterial infection in children who present to the emergency department with hyperpyrexia (temperature of 106 degrees F or higher). Pediatrics.

[43] McCarthy P.L., Dolan T.F. (1976). Hyperpyrexia in children. Eight-year emergency room experience. Am. J. Dis. Child..

[44] World Health Organization (1990). Acute Respiratory Infections in Children: Case Management in Small Hospitals in Developing Countries.

[45] Shah S., Bachur R., Kim D., Neuman M.I. (2010). Lack of predictive value of tachypnea in the diagnosis of pneumonia in children. Pediatr. Infect. Dis. J..

[46] Nijman R.G., Thompson M., van Veen M., Perera R., Moll H.A., Oostenbrink R. (2012). Derivation and validation of age and temperature specific reference values and centile charts to predict lower respiratory tract infection in children with fever: Prospective observational study. BMJ.

[47] Brent A.J., Lakhanpaul M., Ninis N., Levin M., MacFaul R., Thompson M. (2011). Evaluation of temperature-pulse centile charts in identifying serious bacterial illness: Observational cohort study. Arch. Dis. Child..

[48] Shacham S., Kozer E., Bahat H., Mordish Y., Goldman M. (2009). Bulging fontanelle in febrile infants: Is lumbar puncture mandatory?. Arch. Dis. Child..

[49] Mandl K.D., Stack A.M., Fleisher G.R. (1997). Incidence of bacteremia in infants and children with fever and petechiae. J. Pediatr..

[50] Brogan P.A., Raffles A. (2000). The management of fever and petechiae: Making sense of rash decisions. Arch. Dis. Child..

[51] Tal Y., Even L., Kugelman A., Hardoff D., Srugo I., Jaffe M. (1997). The clinical significance of rigors in febrile children. Eur. J. Pediatr..

[52] Thompson M.J., Ninis N., Perera R., Mayon-White R., Phillips C., Bailey L., Harnden A., Mant D., Levin M. (2006). Clinical recognition of meningococcal disease in children and adolescents. Lancet.

[53] So H.K., Li A.M., Au C.T., Zhang J., Lau J., Fok T.F., Wing Y.K. (2012). Night sweats in children: Prevalence and associated factors. Arch. Dis. Child..

[54] Van den Bruel A., Thompson M., Buntinx F., Mant D. (2012). Clinicians’ gut feeling about serious infections in children: Observational study. BMJ.

[55] American Academy of Pediatrics (2016). Pediatric Education for Prehospital Professionals.

[56] Dieckmann R.A., Brownstein D., Gausche-Hill M. (2010). The pediatric assessment triangle: A novel approach for the rapid evaluation of children. Pediatr. Emerg. Care.

[57] Greenhow T.L., Hung Y.-Y., Herz A.M., Losada E., Pantell R.H. (2014). The Changing Epidemiology of Serious Bacterial Infections in Young Infants. Pediatr. Infect. Dis. J..

[58] Huppler A.R., Eickhoff J.C., Wald E.R. (2010). Performance of low-risk criteria in the evaluation of young infants with fever: Review of the literature. Pediatrics.

[59] Aronson P., Thurm C., Alpern E., Alessandrini E., Williams D., Shah S., Nigrovic L., McCulloh R., Schondelmeyer A., Tieder J. (2014). Variation in care of the febrile young infant. Pediatrics.

[60] NICE (National Institute for Health and Clinical Excellence) (2015). Bronchiolitis in Children: Diagnosis and Management.

[61] Ralston S.L., Lieberthal A.S., Meissner H.C., Alverson B.K., Baley J.E., Gadomski A.M., Johnson D.W., Light M.J., Maraqa N.F., Mendonca E.A. (2014). American Academy of Pediatrics Clinical practice guideline: The diagnosis, management, and prevention of bronchiolitis. Pediatrics.

[62] Levine D.A., Platt S.L., Dayan P.S., Macias C.G., Zorc J.J., Krief W., Schor J., Bank D., Fefferman N., Shaw K.N. (2004). Multicenter RSV-SBI Study Group of the Pediatric Emergency Medicine Collaborative Research Committee of the American Academy of Pediatrics Risk of serious bacterial infection in young febrile infants with respiratory syncytial virus infections. Pediatrics.

[63] Yo C.-H., Hsieh P.-S., Lee S.-H., Wu J.-Y., Chang S.-S., Tasi K.-C., Lee C.-C. (2012). Comparison of the Test Characteristics of Procalcitonin to C-Reactive Protein and Leukocytosis for the Detection of Serious Bacterial Infections in Children Presenting With Fever Without Source: A Systematic Review and Meta-analysis. Ann. Emerg. Med..

[64] Van den Bruel A., Thompson M.J., Haj-Hassan T., Stevens R., Moll H., Lakhanpaul M., Mant D. (2011). Diagnostic value of laboratory tests in identifying serious infections in febrile children: Systematic review. BMJ.

[65] Milcent K., Faesch S., Gras-Le Guen C., Dubos F., Poulalhon C., Badier I., Marc E., Laguille C., de Pontual L., Mosca A. (2015). Use of Procalcitonin Assays to Predict Serious Bacterial Infection in Young Febrile Infants. JAMA Pediatr..

[66] Luaces-Cubells C., Mintegi S., García-García J.-J., Astobiza E., Garrido-Romero R., Velasco-Rodríguez J., Benito J. (2012). Procalcitonin to Detect Invasive Bacterial Infection in Non-Toxic-appearing Infants with Fever Without Apparent Source in the Emergency Department. Pediatr. Infect. Dis. J..

[67] Mace S.E., Gemme S.R., Valente J.H., Eskin B., Bakes K., Brecher D., Rn M.S.N., Cen A.P.N.A., Brown M.D., College A. (2016). Clinical Policy for Well-Appearing Infants and Children Younger Than 2 Years of Age Presenting to the Emergency Department With Fever. Ann. Emerg. Med..

[68] Martin N.G., Sadarangani M., Pollard A.J., Goldacre M.J. (2014). Hospital admission rates for meningitis and septicaemia caused by Haemophilus influenzae, Neisseria meningitidis, and Streptococcus pneumoniae in children in England over five decades: A population-based observational study. Lancet Infect. Dis..

[69] Haddon R.A., Barnett P.L., Grimwood K., Hogg G.G. (1999). Bacteraemia in febrile children presenting to a paediatric emergency department. Med. J. Aust..

[70] Bressan S., Berlese P., Mion T., Masiero S., Cavallaro A., Da Dalt L. (2012). Bacteremia in feverish children presenting to the emergency department: A retrospective study and literature review. Acta Paediatr..

[71] Simon A.E., Lukacs S.L., Mendola P. (2013). National trends in emergency department use of urinalysis, complete blood count, and blood culture for fever without a source among children aged 2 to 24 months in the pneumococcal conjugate vaccine 7 era. Pediatr. Emerg. Care.

[72] Waight P.A., Andrews N.J., Ladhani S.N., Sheppard C.L., Slack M.P.E., Miller E. (2015). Effect of the 13-valent pneumococcal conjugate vaccine on invasive pneumococcal disease in England and Wales 4 years after its introduction: An observational cohort study. Lancet Infect. Dis..

[73] Simonsen L., Taylor R.J., Schuck-Paim C., Lustig R., Haber M., Klugman K.P. (2014). Effect of 13-valent pneumococcal conjugate vaccine on admissions to hospital 2 years after its introduction in the USA: A time series analysis. Lancet Respir. Med..

[74] Thomas M.F., Sheppard C.L., Guiver M., Slack M.P.E., George R.C., Gorton R., Paton J.Y., Simmister C., Cliff D., Elemraid M.A. (2012). Emergence of pneumococcal 19A empyema in UK children. Arch. Dis. Child..

[75] Strachan R.E., Snelling T.L., Jaffé A. (2013). Increased paediatric hospitalizations for empyema in Australia after introduction of the 7-valent pneumococcal conjugate vaccine. Bull. World Health Organ..

[76] Saxena S., Atchison C., Cecil E., Sharland M., Koshy E., Bottle A. (2015). Additive impact of pneumococcal conjugate vaccines on pneumonia and empyema hospital admissions in England. J. Infect..

[77] Shaikh N., Morone N.E., Bost J.E., Farrell M.H. (2008). Prevalence of Urinary Tract Infection in Childhood: A Meta-Analysis. Pediatr. Infect. Dis..

[78] Subcommittee on Urinary Tract Infection Steering Committee on Quality Improvement and Management (2011). Urinary tract infection: Clinical practice guideline for the diagnosis and management of the initial UTI in febrile infants and children 2 to 24 months. Pediatrics.

[79] Simon A.E., Lukacs S.L., Mendola P. (2011). Emergency Department Laboratory Evaluations of Fever Without Source in Children Aged 3 to 36 Months. Pediatrics.

[80] Ramos-Jorge J., Pordeus I.A., Ramos-Jorge M.L., Paiva S.M. (2011). Prospective Longitudinal Study of Signs and Symptoms Associated With Primary Tooth Eruption. Pediatrics.

[81] Prymula R., Habib A., François N., Borys D., Schuerman L. (2013). Immunological memory and nasopharyngeal carriage in 4-year-old children previously primed and boosted with 10-valent pneumococcal non-typeable Haemophilus influenzae protein D conjugate vaccine (PHiD-CV) with or without concomitant prophylactic paracetamo. Vaccine.

[82] Das R.R., Panigrahi I., Naik S.S. (2014). The Effect of Prophylactic Antipyretic Administration on Post-Vaccination Adverse Reactions and Antibody Response in Children: A Systematic Review. PLoS ONE.

[83] Dagan R., Powell K.R., Hall C.B., Menegus M.A. (1985). Identification of infants unlikely to have serious bacterial infection although hospitalized for suspected sepsis. J. Pediatr..

[84] Baker M.D., Bell L.M., Avner J.R. (1993). Outpatient management without antibiotics of fever in selected infants. N. Engl. J. Med..

[85] Baskin M.N., O’Rourke E.J., Fleisher G.R. (1992). Outpatient treatment of febrile infants 28 to 89 days of age with intramuscular administration of ceftriaxone. J. Pediatr..

[86] Thompson M., Van den Bruel A., Verbakel J., Lakhanpaul M., Haj-Hassan T., Stevens R., Moll H., Buntinx F., Berger M., Aertgeerts B. (2012). Systematic review and validation of prediction rules for identifying children with serious infections in emergency departments and urgent-access primary care. Health Technol. Assess..

[87] Verbakel J.Y., Van den Bruel A., Thompson M., Stevens R., Aertgeerts B., Oostenbrink R., Moll H.A., Berger M.Y., Lakhanpaul M., Mant D. (2013). How well do clinical prediction rules perform in identifying serious infections in acutely ill children across an international network of ambulatory care datasets?. BMC Med..

[88] Kerkhof E., Lakhanpaul M., Ray S., Verbakel J.Y., Van den Bruel A., Thompson M., Berger M.Y., Moll H.A., Oostenbrink R. (2014). The Predictive Value of the NICE “Red Traffic Lights” in Acutely Ill Children. PLoS ONE.

[89] Craig J.C., Williams G.J., Jones M., Codarini M., Macaskill P., Hayen A., Irwig L., Fitzgerald D.A., Isaacs D., McCaskill M. (2010). The accuracy of clinical symptoms and signs for the diagnosis of serious bacterial infection in young febrile children: Prospective cohort study of 15,781 febrile illnesses. BMJ.

[90] De Vos-Kerkhof E., Nijman R.G., Vergouwe Y., Polinder S., Steyerberg E.W., van der Lei J., Moll H.A., Oostenbrink R. (2015). Impact of a Clinical Decision Model for Febrile Children at Risk for Serious Bacterial Infections at the Emergency Department: A Randomized Controlled Trial. PLoS ONE.

[91] De Vos-Kerkhof E., Geurts D.H., Wiggers M., Moll H.A., Oostenbrink R. (2015). Tools for “safety netting” in common paediatric illnesses: A systematic review in emergency care. Arch. Dis. Child..

[92] Vaillancourt S., Guttmann A., Li Q., Chan I.Y.M., Vermeulen M.J., Schull M.J. (2015). Repeated Emergency Department Visits Among Children Admitted With Meningitis or Septicemia: A Population-Based Study. Ann. Emerg. Med..

[93] Green S.M., Nigrovic L.E., Krauss B.S. (2015). Sick Kids Look Sick. Ann. Emerg. Med..

[94] Liu L., Oza S., Hogan D., Perin J., Rudan I., Lawn J.E., Cousens S., Mathers C., Black R.E. (2015). Global, regional, and national causes of child mortality in 2000–2013, with projections to inform post-2015 priorities: An updated systematic analysis. Lancet.

[95] Le Doare K., Nichols A.-L., Payne H., Wells R., Navidnia S., Appleby G., Calton E., Sharland M., Ladhani S.N. (2014). Very low rates of culture-confirmed invasive bacterial infections in a prospective 3-year population-based surveillance in Southwest London. Arch. Dis. Child..

[96] Brennan C., Wang V.J. (2015). Management of Fever and Suspected Infection in Pediatric Patients with Central Venous Catheters. Pediatr. Emerg. Med. Pract..

[97] McCrea N., O’Donnell R., Brown R. (2013). Outpatient respiratory management of the child with severe neurological impairment. Arch. Dis. Child. Educ. Pract. Ed..

[98] Smith M., Peacock G., Uyeki T.M., Moore C. (2015). Influenza vaccination in children with neurologic or neurodevelopmental disorders. Vaccine.

[99] Fahimzad A., Babaie D., Ghoroubi J., Zahed G., Rafiei Tabatabaei S. (2013). Common Infections among Disabled Children Admitted to Hospital. Arch. Pediatr. Infect. Dis..

[100] Gündoğdu G., Kömür M., Avlan D., Sarı F.B., Delibaş A., Taşdelen B., Naycı A., Okuyaz Ç. (2013). Relationship of bladder dysfunction with upper urinary tract deterioration in cerebral palsy. J. Pediatr. Urol..

[101] Anígilájé E.A., Bitto T.T. (2013). Prevalence and predictors of urinary tract infections among children with cerebral palsy in Makurdi, Nigeria. Open J. Pediatr..

[102] Elkaiali L., Ratliff K., Oueis H. (2016). Dental Treatment Considerations for Children with Complex Medical Histories: A Case of Townes-Brock Syndrome. J. Mich. Dent. Assoc..

[103] Young N.L., McCormick A.M., Gilbert T., Ayling-Campos A., Burke T., Fehlings D., Wedge J. (2011). Reasons for Hospital Admissions Among Youth and Young Adults With Cerebral Palsy. Arch. Phys. Med. Rehabil..

[104] Deda G., Çaksen H., Çiftçi E., İnce E., Doğru Ü. (2002). Very high serum creatine kinase level in a child with dyskinetic cerebral palsy. Brain Dev..

[105] Frampton A., Spinks J. (2005). Hyperthermia associated with central anticholinergic syndrome caused by a transdermal hyoscine patch in a child with cerebral palsy. Emerg. Med. J..

[106] Specchio N., Carotenuto A., Trivisano M., Cappelletti S., Vigevano F., Fusco L. (2011). Prolonged episode of dystonia and dyskinesia resembling status epilepticus following acute intrathecal baclofen withdrawal. Epilepsy Behav..

[107] Choi S.W., Park S.-W., Kwon Y.S., Oh I.S., Lim M.K., Kim W.H., Suh C.H. (2007). MR imaging in a child with scurvy: A case report. Korean J. Radiol..

[108] Massaro M., Pastore S., Ventura A., Barbi E. (2013). Pain in cognitively impaired children: A focus for general pediatricians. Eur. J. Pediatr..

[109] Bray G.P., Harrison P.M., O’Grady J.G., Tredger J.M., Williams R. (1992). Long-term anticonvulsant therapy worsens outcome in paracetamol-induced fulminant hepatic failure. Hum. Exp. Toxicol..

[110] Grüber C., Keil T., Kulig M., Roll S., Wahn U., Wahn V., Groeger M., Zepp F., Bieber I., Forster J. (2008). History of respiratory infections in the first 12 yr among children from a birth cohort. Pediatr. Allergy Immunol..

[111] Lankisch P., Schiffner J., Ghosh S., Babor F., Borkhardt A., Laws H.-J. (2015). The Duesseldorf Warning Signs for Primary Immunodeficiency: Is it Time to Change the Rules?. J. Clin. Immunol..

[112] Gattorno M., Caorsi R., Meini A., Cattalini M., Federici S., Zulian F., Cortis E., Calcagno G., Tommasini A., Consolini R. (2009). Differentiating PFAPA syndrome from monogenic periodic fevers. Pediatrics.

[113] Shohat M., Pagon R.A., Adam M.P., Ardinger H.H. (1993). Familial Mediterranean Fever.

[114] Blüschke G., Nüsken K.D., Schneider H. (2010). Prevalence and prevention of severe complications of hypohidrotic ectodermal dysplasia in infancy. Early Hum. Dev..

[115] Zizzo C., Colomba P., Albeggiani G., Gallizzi R., Iemolo F., Nuzzo D., Vasto S., Caruso C., Duro G. (2013). Misdiagnosis of familial Mediterranean fever in patients with Anderson-Fabry disease. Clin. Genet..

[116] Chow A., Robinson J.L. (2011). Fever of unknown origin in children: A systematic review. World J. Pediatr..

[117] Chandrakasan S., Filipovich A.H. (2013). Hemophagocytic lymphohistiocytosis: Advances in pathophysiology, diagnosis, and treatment. J. Pediatr..

[118] Henter J.-I., Horne A., Aricó M., Egeler R.M., Filipovich A.H., Imashuku S., Ladisch S., McClain K., Webb D., Winiarski J. (2007). HLH-2004: Diagnostic and therapeutic guidelines for hemophagocytic lymphohistiocytosis. Pediatr. Blood Cancer.

[119] Alvar J., Vélez I.D., Bern C., Herrero M., Desjeux P., Cano J., Jannin J., den Boer M. (2012). Leishmaniasis Worldwide and Global Estimates of Its Incidence. PLoS ONE.

[120] Rigante D., Rossodivita A., Cantarini L. (2014). Unmasking an obstinate fever. Isr. Med. Assoc. J..

[121] Flaherty E.G., MacMillan H.L. (2013). Caregiver-Fabricated Illness in a Child: A Manifestation of Child Maltreatment. Pediatrics.

[122] Patel R.A., Gallagher J.C. (2010). Drug fever. Pharmacotherapy.

[123] (2015). Centers for Disease Control and Prevention CDC Health Information for International Travel. http://wwwnc.cdc.gov/travel/page/yellowbook-home.

[124] Sahm L.J., Kelly M., McCarthy S., O’Sullivan R., Shiely F., Romsing J. (2015). Knowledge, attitudes and beliefs of parents regarding fever in children: A Danish interview study. Acta Paediatr..

[125] Poirier M.P., Collins E.P., McGuire E. (2010). Fever phobia: A survey of caregivers of children seen in a pediatric emergency department. Clin. Pediatr..

[126] Richardson M., Purssell E. (2015). Who’s afraid of fever?. Arch. Dis. Child..

[127] Greensmith L. (2013). Nurses’ knowledge of and attitudes towards fever and fever management in one Irish children’s hospital. J. Child Health Care.

[128] Schmitt B.D. (1980). Fever phobia: Misconceptions of parents about fevers. Am. J. Dis. Child..

[129] Haveman J., Sminia P., Wondergem J., van der Zee J., Hulshof M.C.C.M. (2005). Effects of hyperthermia on the central nervous system: What was learnt from animal studies?. Int. J. Hyperth..

[130] Teagle A.R., Powell C.V.E. (2014). Is fever phobia driving inappropriate use of antipyretics?. Arch. Dis. Child..

[131] Harden L.M., Kent S., Pittman Q.J., Roth J. (2015). Fever and sickness behavior: Friend or foe?. Brain Behav. Immun..

[132] Purssell E., While A.E. (2013). Does the use of antipyretics in children who have acute infections prolong febrile illness? A systematic review and meta-analysis. J. Pediatr..

[133] Strengell T., Uhari M., Tarkka R., Uusimaa J., Alen R., Lautala P., Rantala H. (2009). Antipyretic agents for preventing recurrences of febrile seizures: Randomized controlled trial. Arch. Pediatr. Adolesc. Med..

[134] King D. (2013). Question 2: Does a failure to respond to antipyretics predict serious illness in children with a fever?. Arch. Dis. Child..

[135] Sullivan J.E., Farrar H.C, American Academy of Pediatrics Section on Clinical Pharmacology and Therapeutics, Committee on Drugs (2011). Fever and antipyretic use in children. Pediatrics.

[136] Parikh S., Saneto R., Falk M.J., Anselm I., Cohen B.H., Haas R., Medicine Society T.M. (2009). A modern approach to the treatment of mitochondrial disease. Curr. Treat. Opt. Neurol..

[137] De Caen A.R., Berg M.D., Chameides L., Gooden C.K., Hickey R.W., Scott H.F., Sutton R.M., Tijssen J.A., Topjian A., van der Jagt É.W. (2015). Part 12: Pediatric Advanced Life Support: 2015 American Heart Association Guidelines Update for Cardiopulmonary Resuscitation and Emergency Cardiovascular Care. Circulation.

[138] De Martino M., Chiarugi A. (2015). Recent Advances in Pediatric Use of Oral Paracetamol in Fever and Pain Management. Pain Ther..

[139] Wong T., Stang A.S., Ganshorn H., Hartling L., Maconochie I.K., Thomsen A.M., Johnson D.W. (2014). Combined and alternating paracetamol and ibuprofen therapy for febrile children. Evid. Based Child Heal. A Cochrane Rev. J..

[140] Yue Z., Jiang P., Sun H., Wu J. (2014). Association between an excess risk of acute kidney injury and concomitant use of ibuprofen and acetaminophen in children, retrospective analysis of a spontaneous reporting system. Eur. J. Clin. Pharmacol..

[141] Emmerton L., Chaw X.Y., Kelly F., Kairuz T., Marriott J., Wheeler A., Moles R. (2014). Management of children’s fever by parents and caregivers: Practical measurement of functional health literacy. J. Child. Health Care.

[142] Heubi J.E., Barbacci M.B., Zimmerman H.J. (1998). Therapeutic misadventures with acetaminophen: Hepatoxicity after multiple doses in children. J. Pediatr..

[143] Marzuillo P., Guarino S., Barbi E. (2014). Paracetamol: A focus for the general pediatrician. Eur. J. Pediatr..

[144] Rajanayagam J., Bishop J.R., Lewindon P.J., Evans H.M. (2015). Paracetamol-associated acute liver failure in Australian and New Zealand children: High rate of medication errors. Arch. Dis. Child..

[145] Cheelo M., Lodge C.J., Dharmage S.C., Simpson J.A., Matheson M., Heinrich J., Lowe A.J. (2015). Paracetamol exposure in pregnancy and early childhood and development of childhood asthma: A systematic review and meta-analysis. Arch. Dis. Child..

